# Protective effect of quercetin against oxidative stress caused by dimethoate in human peripheral blood lymphocytes

**DOI:** 10.1186/1476-511X-10-149

**Published:** 2011-08-23

**Authors:** Bochra Gargouri, Riadh Ben Mansour, Fatma Ben Abdallah, Abdelfetteh Elfekih, Saloua Lassoued, Hamden Khaled

**Affiliations:** 1 Unité de Biotechnologie et Pathologies, Institut Supérieur de Biotechnologie de Sfax, 3038, Tunisie; 2 Unité de Physiologie Animale, Faculté des Sciences de Sfax, 3037, Tunisie

**Keywords:** Quercetin, Dimethoate, malondialdehyde, thiol, superoxide dismutase, catalase, lymphocytes

## Abstract

**Background:**

The aim of this study is to investigate the effect of quercetin in alleviating the cytotoxic effects of Dimethoate in human peripheral blood lymphocytes.

**Methods:**

Lymphocytes were divided into too groups. The first group, lymphocytes were incubated for 4 h at 37°C with different concentrations (0, 40, 60 and 100 mM) of Dimethoate. The second group was preincubated with quercetin for 30 min and followed by Dim incubation for 4 h at 37°C.

**Results:**

Following in vitro incubation, Dimethoate caused a significant increase in malondialdehyde levels, a significant decrease in thiol levels, as well as a significant increase in superoxide dismutase, and catalase activities in lymphocytes at different concentrations. Quercetin pretreated lymphocytes showed a significant protection against the cytotoxic effects inducted by Dimethoate on the studied parameters.

**Conclusion:**

In conclusion, antioxidant quercetin could protect against Dimethoate-induced oxidative stress by decreasing lipid peroxidation, protein oxidation and increasing superoxide dismutase and catalase activities in human lymphocytes.

## Background

Dimethoate is an insecticide used to kill insects systemically and on contact. It is used against a wide range of insects, on ornamental plants, apples, corn, cotton, grapefruit, grapes, lemons, tobacco, tomatoes, watermelons and other vegetables. Dimethoate is one of a class of insecticides referred to as organophosphates (OP). These chemicals act by interfering with the activities of cholinesterase, an enzyme that is essential for the proper working of the nervous systems of both humans and insects. The number of human poisonings with (OP) pesticides is estimated at around 3,000,000 per year, and the number of deaths and casualties around 200,000 per year [1]. Recent findings indicate that toxic manifestations induced by OP may be associated with the enhanced production of reactive oxygen species (ROS) [2]. In the body, these pesticides can disturb the balance of antioxidants as well as lipid peroxidation [2]. The human cells are continuously attacked by ROS, which arise as natural products of normal cellular machinery energy production and by exhaustive exercise or by chemical agents in the environment. In normal conditions, and in attempt to defend against oxidative stress, human cells are well equipped with several enzymatic and non-enzymatic antioxidants [3]. Among antioxidant enzymes, the superoxide dismutase (SOD) catalyzes dismutation of superoxide to hydrogen peroxide (H_2_O_2_) and molecular oxygen. The decomposition of hydrogen peroxide to nontoxic compounds is the main function of catalase (CAT) [4]. SOD and CAT usually act in a synergetic manner [4]. Cells show various structural alterations and disruptions of the antioxidant defense system following exposure to toxic chemicals and environmental pollutants. Indeed, available reports indicate that the enzyme activities associated with antioxidant defense mechanisms are altered by insecticides both in vivo and in vitro [5].

There is no enough data on the cytotoxic effects of Dim in the human lymphocytes in vitro. Therefore, this study aimed to determine the effect of Dim at several different doses, either alone or in combination with quercetin, in MDA and thiol levels and the activities of SOD and CAT in human lymphocytes.

## Results

### MDA Levels

Four hours of incubation of Dim in different concentrations with human lymphocytes caused a significant increase in MDA levels (p < 0.05) (Table 1). The effects were concentrations dependent. Lymphocytes pretreated with quercetin before incubation with Dim showed a significant decrease in MDA levels as compared to Dim alone-treated lymphocytes (p < 0.05).

**Table 1:** Changes in MDA levels of human lymphocytes incubated with different concentrations of Dimethoate (0, 40, 60,and 100 mM), quercetin (20 μg/ml)

Experimental Groups	MDA mg/mg proteins
Control	2.03 ± 0.1
Quercetin (20 μg/ml)	2.67 ± 0.71#
Dim (40 mM)	3.62 ± 0.22*
Dim (40 mM)+ Quercetin (20 μg/ml)	2.99 ± 0.15#
Dim (60 mM)	4.51 ± 0.44*
Dim (60 mM)+ Quercetin (20 μg/ml)	3.83 ± 0.31#
Dim (100 mM)	5.92 ± 0.17*
Dim (100 mM)+ Quercetin (20 μg/ml)	4.72 ± 0.34#

The values are expressed as means ± SD; n = 20; controls are lymphocytes without Dimethoate, lymphocytes incubated with quercetin (20 μg/ml). # p < 0.05, compared with treatments without quercetin (20 μg/ml), compared with controls (lymphocytes without Dimethoate).

### Protein thiol level

To determine protein oxidation, SH levels were assessed in lymphocytes treated with Dim. Dim treatment caused a decrease in SH levels (p < 0.05) (Table 2). Protein oxidation was significantly inhibited by supplementation of quercetin (Table 2). Our data shows that the inhibitory effect of quercetin on protein oxidation was greater with increasing quercetin concentrations (Table 2).

**Table 2:** Changes in thiol (SH) levels of human lymphocytes incubated with different concentrations of Dimethoate (0, 40, 60 and 100 mM), quercetin (20 μg/ml)

Experimental Groups	SH level mg/mg proteins
Control	4.33 ± 0.1
Quercetin (20 μg/ml)	4.31 ± 0.1#
Dim (40 mM)	2.53 ± 0.32*
Dim (40 mM)+ Quercetin (20 μg/ml)	3.39 ± 0.55#
Dim (60 mM)	5.67 ± 0.2*
Dim (60 mM)+ Quercetin (20 μg/ml)	4.71 ± 0.3#
Dim (100 mM)	6.81 ± 0.12*
Dim (100 mM)+ Quercetin (20 μg/ml)	5.41 ± 0.31#

The values are expressed as means ± SD; n = 20; controls are lymphocytes without Dimethoate, lymphocytes incubated with quercetin (20 μg/ml). # p < 0.05, compared with treatments without quercetin (20 μg/ml), compared with controls (lymphocytes without Dimethoate).

### SOD Activity

The incubation of lymphocytes with different concentrations of Dim resulted in a significant increase in the activity of SOD (p < 0.05). Pretreatment of lymphocytes with quercetin provided significant protection against the higher SOD activity that was induced by different concentrations of Dim (p < 0.05) (Table 3).

**Table 3:** Changes in SOD activity of human lymphocytes incubated with different concentrations of Dimethoate (0, 40, 60, and 100 mM), quercetin (20 μg/ml)

Experimental Groups	SOD % inhibition
Control	3.33 ± 0.33
Quercetin (20 μg/ml)	3.33 ± 0.3#
Dim (40 mM)	33.33 ± 0.21*
Dim (40 mM)+ Quercetin (20 μg/ml)	15.59 ± 0.11#
Dim (60 mM)	36.66 ± 0.23*
Dim (60 mM)+ Quercetin (20 μg/ml)	24.00 ± 0.33#
Dim (100 mM)	44.44 ± 0.44*
Dim (100 mM)+ Quercetin (20 μg/ml)	33.33 ± 0.12#

The values are expressed as means ± SD; n = 20; controls are lymphocytes without Dimethoate, lymphocytes incubated with quercetin (20 μg/ml) # p < 0.05, compared with treatments with quercetin (20 μg/ml). * p < 0.05, compared with controls (lymphocytes without Dimethoate).

### CAT Activity

The activity of CAT in lymphocytes incubated with different concentrations of Dim for 4 h significantly increased (p < 0.05) (Table 4). Lymphocytes pretreated with quercetin before the exposure to Dim showed a significant protection against the augmentation of CAT activity that was induced by different concentration of Dim (p < 0.05) (Table 4).

**Table 4:** Changes in CAT activity of human lymphocytes incubated with different concentrations of Dimethoate (0, 40, 60, and 100 mM), quercetin (20 μg/ml)

Experimental Groups	CAT U/ml
Control	366.44 ± 0.34
Quercetin (20 μg/ml)	360.00 ± 0.41#
Dim (40 mM)	489.99 ± 0.21*
Dim (40 mM)+ Quercetin (20 μg/ml)	396.98 ± 0.1#
Dim (60 mM)	531.11 ± 0.4*
Dim (60 mM)+ Quercetin (20 μg/ml)	461.51 ± 0.1#
Dim (100 mM)	669.19 ± 0.01*
Dim (100 mM)+ Quercetin (20 μg/ml)	570.12 ± 0.27#

The values are expressed as means ± SD; n = 20; controls are lymphocytes without Dimethoate, lymphocytes incubated with quercetin (20 μg/ml) # p < 0.05, compared with treatments with quercetin (20 μg/ml). * p < 0.05, compared with controls (lymphocytes without Dimethoate).

## Discussion

Pesticides affect the membrane integrity and so disturb functions cells [3]. In this study, ours results showed that incubation with different concentrations of Dim produced lipid peroxidation and protein oxidation in human lymphocytes. Moreover, lymphocytes pretreated with quercetin before incubation with Dim decreased lipid peroxidation as well as protein oxidation as compared with Dim-treated lymphocytes. These results indicated that quercetin may have a beneficial role in lowering Dim toxicity. There are no reports to show the protective effect of quercetin on Dim toxicity of human lymphocytes in vitro. However, a protective effect of quercetin against ROS was reported in erythrocytes [6]. In addition, Kitagawa et al reported an inhibitory effect of quercetin in ROS production in erythrocytes [7]. Recently, some investigators reported that OP interferes with a number of biochemical processes such as protein biosynthesis and mitochondrial respiration. Lipid peroxidation has been suggested as one of the molecular mechanisms involved in OP induced toxicity [8]. OP such as Chlorpyrifos-ethyl caused an in vitro increased lipid peroxidation in human cells and the addition of exogenous antioxidants overcame lipid peroxidation induced by chlorpyrifos-ethyl [5]. Moreover, it has been reported that trichlorfon (Organophosphorus insecticides) may have properties to induce oxidative stress [9]. This status was evidenced by a significant increase of MDA levels when it is incubated at 37°C for 60 min in human erythrocytes. Cells are equipped with several biological mechanisms comprising many antioxidant enzymes such as SOD and CAT to defend against intracellular oxidative stress [3]. SOD, the first line of defense against ROS, catalyzes the dismutation of the superoxide anion into hydrogen peroxide. Hydrogen peroxide can then be transformed into H_2_O and O_2 _by CAT, glutathione peroxidise and peroxiredoxins [10,11]. In this study, SOD and CAT activities were significantly increased by different concentrations of Dim. Lymphocytes pre-treated with quercetin providing significant protection against the higher SOD and CAT activities that were induced by different concentrations of Dim. These data show that the induction of antioxidant activities of SOD and CAT, directly mediated by Dim, accelerated the conversion of superoxide radicals to hydrogen peroxide and the decomposition of hydrogen peroxide and probably antioxidant quercetin may directly scavenge free radicals or modulate the biochemical markers of oxidative stress and antioxidant enzymes. In the literature, it has been reported that methidathion [12], and chlorpyrifos-ethyl [5] caused a decrease while diazinon [13] and dimethoate [14] caused an increase in SOD and CAT activities.

In conclusion, Dim has cytotoxic effects on human lymphocytes evidenced by an increasing lipid peroxidation and proteins oxidation, as welle as antioxidants enzymes activities such as SOD and CAT. However, quercetin could effectively ameliorate the Dim-induced oxidative stress to a large extent. Consequently, supplementation of quercetin may act as a protective agent against the toxicity effect of Dimethaote in human lymphocytes.

## Materials and Methods

Chemicals Dimethoate (C5H12NO3PS2) was obtained from sigma. All other chemicals were supplied by Merck (Germany).

### Lymphocyte preparation

10 normal volunteers donor were recruited into the study after obtaining her informed consent. PBL were isolated from heparinised venous blood by sedimentation in Ficoll-hypaque (Sigma, Germany). Cells were washed three times in PBS (phosphate buffered saline) and immediately used for study.

### Experimental Protocol

The first group, lymphocytes were incubated with different concentrations of Dim (0, 40, 60 and 100 mM) at 37°C for 4 h. The second group was preincubated with 20 μg/ml of quercetin [15] for 30 min followed by the same concentrations of Dim for 4 h at 37°C. After incubation, the level of lymphocytes malondialdehyde (MDA) and the activities of SOD and CAT were carried out. One control contained lymphocytes only (without Dim), and two others served as positive controls, containing quercetin.

### MDA Determination

MDA determination was performed by the thiobarbituric acid reactive species assay. Human lymphocytes (30 μl) were diluted in 500 μl distilled water and 2 volumes of thiobarbituric acid (TBA) agent was added (15% trichloroaceticacid, 0.8% TBA, 0.25 N HCl) was added. The mixture was heated at 95°C for 15 min to form MDA-TBA adduct. Optical density (OD) was measured with a spectrophotometer (Biochrom, Libra S32) at 532 nm. Values were reported to a calibration curve of 1,1,3,3-tetraethoxypropane [16].

### Protein thiol levels Determination

Protein thiols were quantified spectrophotometrically using 5,5-dithionitrobenzoic acid (DTNB); 250 ml of freshly prepared 10 mM DTNB in 0.05 M phosphate buffer pH 8, were added to 50 ml of cell lysate in 1200 ml of 0.05 M phosphate buffer. After incubation in the dark for 15 min at room temperature, the release of 5-thiobenzoic acid was quantified by measuring the absorbance at 412 nm and converted to absolute values using N-acetyl cysteine as standard (0-0.1 mM). A correlation coefficient of r2 = 0.999 was obtained. The absorbance of samples lacking DTNB was subtracted to account for the background absorbance at 412 nm. Samples were analysed in duplicate.

### Catalase Activity Determination

Catalase activity was measured as described previously by Aebi [17]. This method is based on the principle that the absorbance at 240 nm decreases because of H_2_O_2 _dismutation. The extinction coefficient of 43.6 L mol-1 cm-1 for H_2_O_2 _was used for calculation. One unit is defined as the amount of H_2_O_2 _converted into H_2_O and 1/2 O_2 _in 1 min under standard conditions, and the specific activity is reported as units per milligram of protein.

### Superoxide Dismutase Activity Determination

SOD activity was determined by spectrophotometry (420 nm) using pyrogallol assay as described previously by Marklund and Marklund [18] and modified as follows: the autoxidation rate of pyrogallol in Tris-cacodylic acid- diethylenetriaminepenta-acetic acid (DTPA) buffer (pH 8-8.2) was determined (A1). Pyrogallol autoxidation was evaluated under the same conditions after addition of 25 μl of supernatant (A2). The inhibition percentage of pyrogallol oxidation was determined using the following formula: % Inhibition = ((A1-A2)/A1) * 100.

### Protein Quantification

Protein levels were determined using protein essay kit Biorad (Bradford) and bovine serum albumin served as the standard [19].

### Statistical Analysis

Data were expressed as mean +/- SD. The one-way analysis of variance (ANOVA) and the Student-Newman-Keuls post hoc test were performed on the data for intergroup comparisons.

Database management and statistical analysis were performed using SPSS (SPSS 11, Chicago, IL) statistical software package.

## Abbreviations

TBA: thiobarbituric acid reactivity; MDA: malondialdehyde; SOD: superoxide dismutase; CAT: catalase; PBL: Peripheral blood lymphocytes; SH: thiol; ROS: Reactive oxygen species; DNPH: 2,4-dinitrophenylhydrazine; OD: Optical density; TBA: Thiobarbituric acid; DTPA: Tris-cacodylic acid- diethylenetriaminepenta-acetic acid; H_2_O_2_: Hydrogen peroxide; PBS: phosphate buffered saline.

## Competing interests

The authors declare that they have no competing interests.

## Authors' contributions

BG and RBM prepared the study design, carried out all the biological studies, analyzed and discussion of the data, and drafted the manuscript. AE helped in correction of the manuscript. FBA carried out some biological assays and helped with the manuscript preparation. SL and HK participated in thestudy design, discussion the data and helped to draft and correction of the manuscript.

All authors have read and approved the final manuscript.
